# Exploring Novel Funding Strategies for Innovative Medical Research: The HORAO Crowdfunding Campaign

**DOI:** 10.2196/19715

**Published:** 2020-11-11

**Authors:** Philippe Schucht, Diana M Roccaro-Waldmeyer, Michael Murek, Irena Zubak, Johannes Goldberg, Stephanie Falk, Fried-Michael Dahlweid, Andreas Raabe

**Affiliations:** 1 Department of Neurosurgery Inselspital, Bern University Hospital University of Bern Bern Switzerland; 2 Insel Data Science Center Inselspital, Bern University Hospital University of Bern Bern Switzerland; 3 Directorate of Technology and Innovation Inselspital, Bern University Hospital University of Bern Bern Switzerland; 4 DXC Technology Tysons, VA United States

**Keywords:** science funding, crowdfunding, neurosurgery, neurosciences, brain tumor

## Abstract

**Background:**

The rise of the internet and social media has boosted online crowdfunding as a novel strategy to raise funds for kick-starting projects, but it is rarely used in science.

**Objective:**

We report on an online crowdfunding campaign launched in the context of the neuroscience project HORAO. The aim of HORAO was to develop a noninvasive real-time method to visualize neuronal fiber tracts during brain surgery in order to better delineate tumors and to identify crucial cerebral landmarks. The revenue from the crowdfunding campaign was to be used to sponsor a crowdsourcing campaign for the HORAO project.

**Methods:**

We ran a 7-week reward-based crowdfunding campaign on a national crowdfunding platform, offering optional material and experiential rewards in return for a contribution toward raising our target of Swiss francs (CHF) 50,000 in financial support (roughly equivalent to US $50,000 at the time of the campaign). We used various owned media (websites and social media), as well as earned media (press releases and news articles) to raise awareness about our project.

**Results:**

The production of an explanatory video took 60 hours, and 31 posts were published on social media (Facebook, Instagram, and Twitter). The campaign raised a total of CHF 69,109. Approximately half of all donations came from donors who forwent a reward (CHF 28,786, 48.74%); the other half came from donors who chose experiential and material rewards in similar proportions (CHF 14,958, 25.33% and CHF 15,315.69, 25.93%, respectively). Of those with an identifiable relationship to the crowdfunding team, patients and their relatives contributed the largest sum (CHF 17,820, 30.17%), followed by friends and family (CHF 9288, 15.73%) and work colleagues (CHF 6028, 10.21%), while 43.89% of funds came from donors who were either anonymous or had an unknown relationship to the crowdfunding team. Patients and their relatives made the largest donations, with a median value of CHF 200 (IQR 90).

**Conclusions:**

Crowdfunding proved to be a successful strategy to fund a neuroscience project and to raise awareness of a specific clinical problem. Focusing on potential donors with a personal interest in the issue, such as patients and their relatives in our project, is likely to increase funding success. Compared with traditional grant applications, new skills are needed to explain medical challenges to the crowd through video messages and social media.

## Introduction

In 1897, the French scientist Gaston Contremoulins was receiving insufficient support for his work from governmental agencies and therefore turned to the crowd via the popular French newspaper “*Le temps*” to ask for financial help. In what was essentially the first crowdfunded neuroscience project, he and his team raised enough capital to perform the first stereotactic surgery in humans [1]. More than a century later, in response to the financial crisis of 2007 and 2008, and thanks to the rise of the internet and e-commerce, crowdfunding has emerged as an alternative method to raise funds and has been gaining popularity ever since [2,3]. Especially for high-risk projects, which are rarely supported by traditional funding agencies [4,5], crowdfunding represents a potentially more promising alternative. Crowdfunding, a term first coined in 2007 [6], can be defined as an innovative method of fundraising for a project or business, which typically involves a large number of rather small contributions from individuals following an open call through the internet [7,8]. In the health care domain, the competition for medical research funding from government sources, such as the National Institutes of Health, is continuously increasing [4,5,9], making crowdfunding an attractive alternative for clinical and scientific research funding. Unlike traditional funding mechanisms, it does not involve a rigorous peer-review process, but instead offers a way to reach more diverse audiences and to raise public awareness about scientific problems [5,10,11].

From an economic perspective, the following four different business models of crowdfunding can be distinguished, depending on what donors receive in return for their financial contributions: donation-based crowdfunding (no return on investment expected), reward-based crowdfunding (optional nonmonetary returns), lending-based crowdfunding (return of the funds, possibly with interest), and equity-based crowdfunding (future profit of the venture is shared) [7-9,12]. Crowdfunding campaigns aiming to foster medical research are often reward-based [9]. Although reward-based crowdfunding can involve prepurchasing a newly developed product, it is understood here as donating in return for nonfinancial potentially experiential rewards of little economic, but rather symbolic, nontradable value [8,9].

Since the model requires a two-sided market, almost all instances of internet-based crowdfunding fundamentally depend on crowdfunding platforms that act as intermediaries linking fundraisers to potential funders [7,13]. These platforms are essential to create a trustworthy environment in which donors feel secure enough to exchange money for rewards [14]. They also provide a space to describe the project, facilitate communication with potential donors, and help to run the campaign while ensuring standardized processes [7].

During brain tumor surgery, distinguishing tumor tissue from the surrounding healthy tissue remains a challenge. An ideal technology would noninvasively and reproducibly visualize tumor tissue, without time loss in real-time and without harming the surrounding brain. Although technologies, such as 5-aminolevulinic acid fluorescence, are useful to visualize a subset of tumors during surgery [15], as yet it has not been possible to develop a technology applicable to all intrinsic tumors of the brain. We therefore decided to shift the focus from direct identification of tumor tissue to visualization of the microstructure of the brain. Fiber tracts are a hallmark of the white matter of the brain and cannot be seen in tumor tissue. A technology that identifies fiber tracts during surgery would thus allow the surgeon to differentiate between tumor tissue and healthy brain. In order to catalyze interdisciplinary research in this field and to foster the development of an optical instrument that interfaces with current state-of-the-art microscopes to improve the visualization of brain tumor boundaries, we initiated a global, crowdsourced, scientific competition. The aim of the crowdfunding campaign described here was to raise the prize money as an incentive for the above-mentioned crowdsourcing competition.

To our knowledge, this is the first report on an internet-based crowdfunding campaign for clinical neuroscience to be published in a medical journal. Here, we describe our experience with crowdfunding to finance a neuroscience project and analyze its strengths and the challenges.

## Methods

### Crowdfunding Campaign

**Figure 1:**
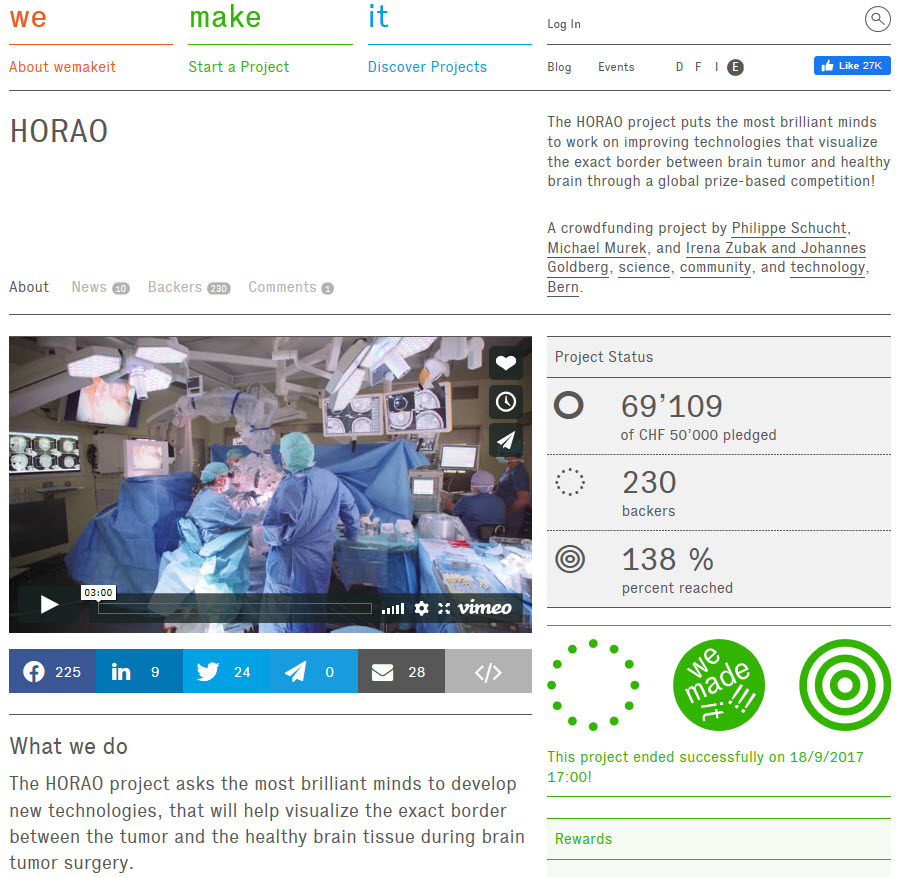
Screenshot taken from the online donation platform at the time of completion of the HORAO campaign.

The campaign was launched on the Science Booster Channel of wemakeit, a leading Swiss crowdfunding platform, on August 3, 2017 [16] (Figure 1). The funding target was set at 50,000 Swiss francs (CHF; roughly equivalent to US $50,000 at the time of the campaign), and the duration of the campaign was 47 days. Depending on how much they donated, donors could choose from 12 different rewards or decide to forgo the reward. Material rewards included project t-shirts, thank-you cards and plasters designed explicitly for this campaign, a bestseller novel signed by the team of physicians, and a unique work of art created exclusively for the purpose of this campaign that was donated by the artist. Experiences on offer included a visit to an artist’s studio, an invitation to the scientific conference to be held at the end of the crowdsourcing competition, a guided tour of the neurosurgical department, neurosurgical training in the skills lab, a one-day visit to the neurosurgical department with the opportunity to shadow physicians, and a dinner with the team of physicians. Donors were classified according to their relationship to the crowdfunding team as (1) friends and family members, (2) work colleagues, (3) patients (and their relatives), (4) unknown, and (5) anonymous.

### Media Activity

Owned (websites and social media) and earned media (press releases and news articles) were used to raise public awareness about the campaign. A website was created specifically for the purpose of this crowdfunding and the subsequent crowdsourcing campaign [17]. A 3-minute explanatory video was produced and published on wemakeit, as well as on the YouTube channel of our hospital group (Multimedia Appendix 1) [18]. The video featured three brain tumor patients and had an overall production time of 60 hours. Involvement of social media required daily attention. A total of 31 posts and tweets were published on Facebook, Instagram, and Twitter during the course of the campaign. Two press releases on August 03 and September 19, 2017, resulted in the publication of 14 media reports (print, online, and radio) at the beginning (eight reports on August 03 and six reports on August 04), and nine media reports (print and online) at the end of the campaign. During the campaign, a report was printed in the largest Swiss newspaper *20 Minuten* (on August 08, 2017), supplemented by a sponsored short article that continued to be featured in the electronic version of the newspaper throughout the week of August 18 to August 24 (a pro-bono donation made by the news portal). A contribution to the newsletter of the European Association of Neurosurgical Societies was also submitted. Finally, regular updates were published on the websites of our hospital group, our university hospital, our neurosurgical department, and our cancer center.

### Statistical Analysis

IBM SPSS Statistics Version 25 (IBM Corp) was used for all statistical analyses and for the creation of the graphs. A Kruskal-Wallis H test was performed to compare total donations per day across the seven campaign weeks, as well as to compare the size of individual donations between donor groups with different relationships to the crowdfunding team. Bonferroni correction was used to adjust for multiple post-hoc comparisons. A Mann-Whitney *U* test was performed to investigate the effect of publicity in the newspaper *20 Minuten* (August 08 to August 17 vs August 19 to August 25). A Pearson chi-squared test was performed to assess the correlation between reward type and type of relationship, while Goodman and Kruskal tau served to determine the extent of the bidirectional interdependence. Statistical significance was set at *P*<.05.

## Results

**Figure 2:**
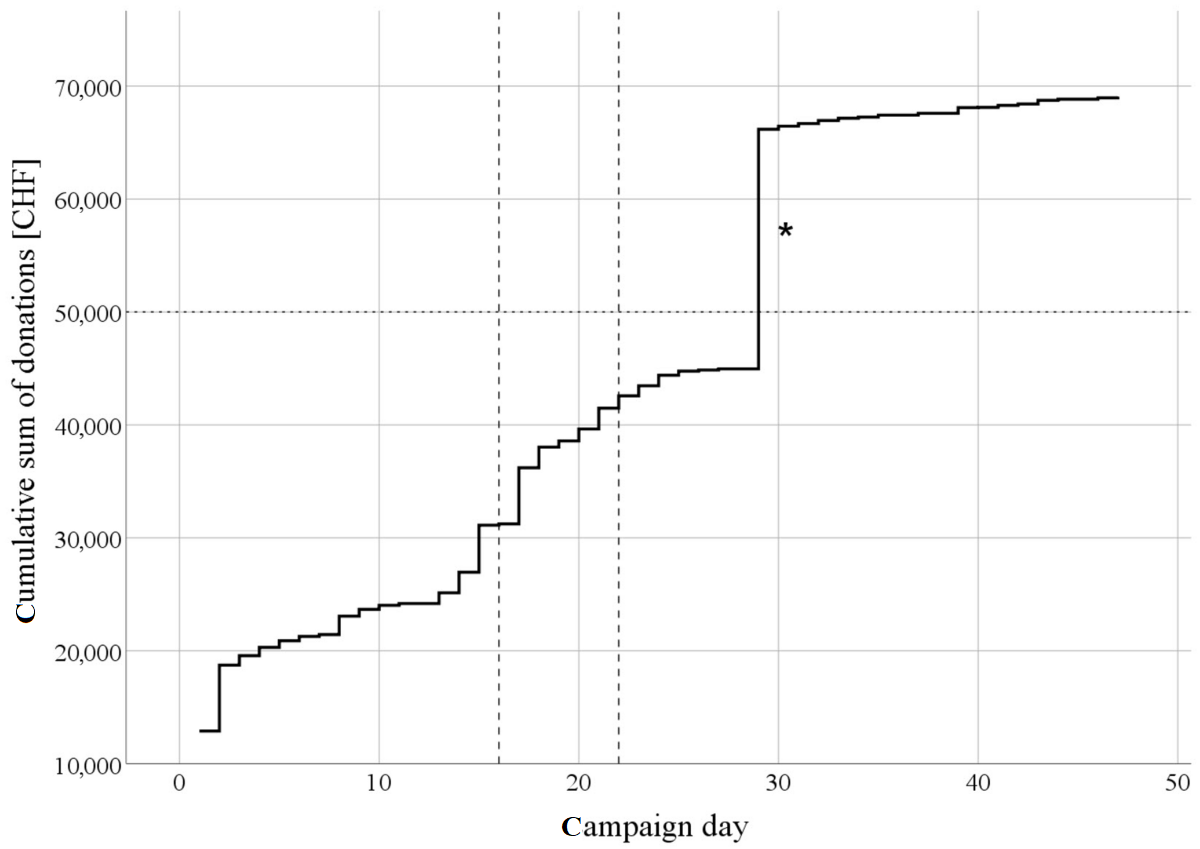
Cumulative sum of daily donations across the 7 weeks of the HORAO campaign. The dotted horizontal line indicates the funding goal of Swiss francs (CHF) 50,000 was reached on day 30, triggering the contribution of an additional CHF 10,000 by Gebert Rüf Stiftung (asterisk). The dashed vertical lines delimit the 7-day period during which an article on the project was featured in the electronic version of the newspaper *20 Minuten* (days 16-22 of the campaign).

On campaign day 30 (August 31, 2017), the funding goal of CHF 50,000 was reached (Figure 2). The crowdfunding campaign ended after the predefined duration of 47 days. A total of CHF 69,109 (contributed by 235 donors) was raised, exceeding the target of CHF 50,000 by 38.22%. Our expenditures were as follows: CHF 6911 for the crowdfunding platform (10.00% of the total revenue), CHF 4839 for the production of the video, CHF 1736 for the purchase of the material rewards, and CHF 280 for web services, resulting in a total revenue of CHF 55,443. Of this total, CHF 50,000 was used as prize money for the crowdsourcing campaign, and the rest (CHF 5,443) was used for the organization of the final crowdsourcing conference, during which the finalists described their solution to the public and competed for the prize.

Our crowdfunding campaign received CHF 10,000 in additional support from the Science Booster Channel of wemakeit thanks to a generous grant from Gebert Rüf Stiftung (Projekt-Nr. GRS-040/15) upon reaching the predetermined target. This additional prize money has been excluded from the analysis of the funding campaign (ie, 100% always refers to CHF 59,109).

**Figure 3:**
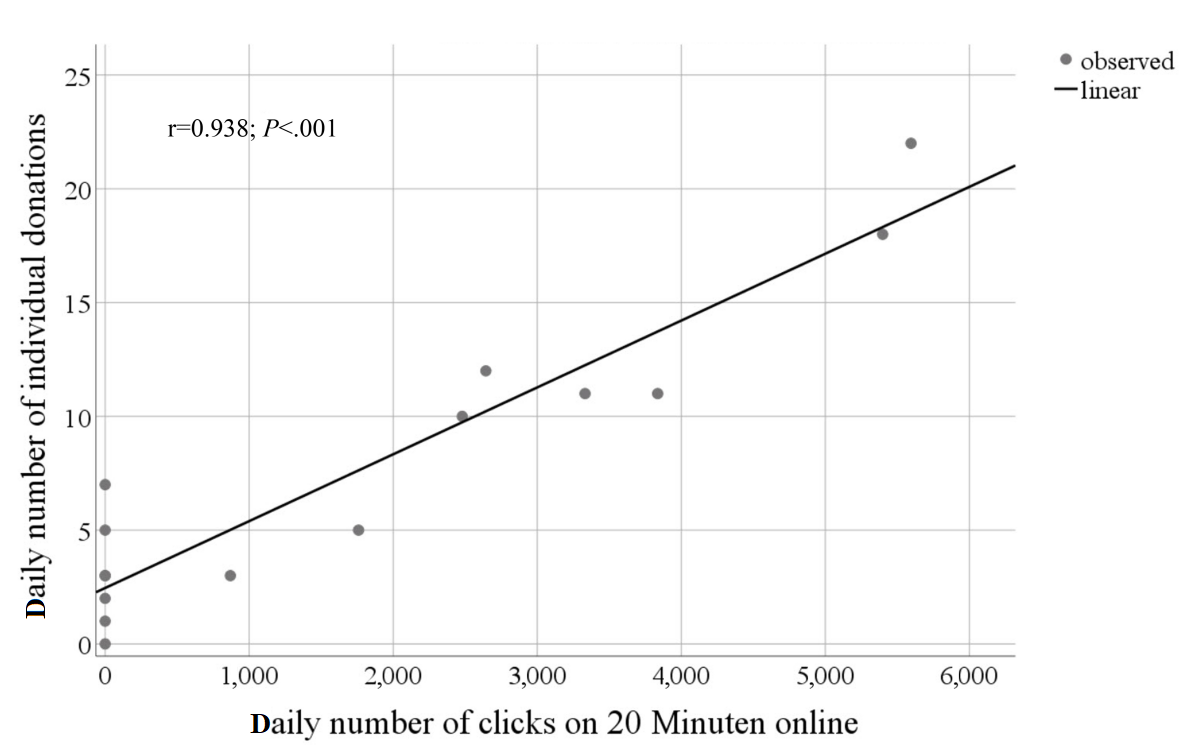
Correlation between the number of clicks per day and the total number of donations made on that day, for the 7 days during which HORAO was featured in the electronic version of the newspaper *20 Minuten* as well as the 7 days before (*r*=0.94, *P*<.001). Points represent individual days. On average, 368 clicks triggered one extra donation (average value of donation: Swiss francs [CHF] 61.40), corresponding to a mean donation of CHF 0.17 per click.

Donations per day tended to be higher in the first four than in the last two weeks of the campaign (Kruskal-Wallis H test: *P*=.03). However, after adjusting for multiple comparisons, none of the differences remained statistically significant. The first week was the most successful (during which 35.84% [CHF 21,185] of the total sum of CHF 59,109 was raised), followed by the fifth week (CHF 12,171, 20.59%) (Table 1). Twenty-two percent of the total sum was raised on day 2 alone, followed by 19% on day 30 and 10% on day 3. On five of the 48 days, no donations were made, and four out of these five days were in the final three weeks. During the 7 days when our project was featured in the print version (August 19) and in the electronic version of the newspaper *20 Minuten* (August 19 to August 25), the sum of total donations was approximately 50% higher (CHF 4173) than during the preceding 7 days (August 11 to August 17; CHF 12,229 vs CHF 8056). Additionally, the number of individual donations per day was higher in the second week (median 11, IQR 8 vs median 3, IQR 4; *P*=.01), although the value of total donations per day did not significantly differ between the two weeks (median CHF 1090, IQR 4426 vs median CHF 600, IQR 1660; *P*=.17). In addition, the contributions from both anonymous donors and those whose relationship to the crowdfunding team was unknown peaked during these 7 days (August 19 to August 25; 55% of total donations). These donors had accounted for only 28% of donations in the 7 days before the publicity in *20 Minuten*. The online version of the article generated a total of 25,911 views (869–5595 views per day). There was a strong correlation between the number of views per day and the total number of donations made on that day (*r*=0.94, *P*<.001) (Figure 3). On average, 368 clicks generated one extra donation (average donation: CHF 61). The news portal offered this online exposure free of charge as a donation, and its commercial value was estimated at approximately CHF 20,000.

**Table 1:** Descriptive statistics of donations per day over the 7 weeks.

Week	Median per day (CHF^a^)	Mean per day (CHF)	Maximum per day (CHF)	Sum per week (CHF) (N=59,109), value (%)
1 (August 03 to August 09)	661	2648	12,598	21,185 (35.84)
2 (August 10 to August 16)	570	610	1631	4272 (7.23)
3 (August 17 to August 23)	560	1432	4970	10,025 (16.96)
4 (August 24 to August 30)	937	1318	4097	9228 (15.61)
5 (August 31 to September 06)	230	1739	11,211	12,171 (20.59)
6 (September 07 to September 13)	125	183	500	1284 (2.17)
7 (September 14 to September 18)	100	129	320	644 (1.09)

^a^CHF: Swiss francs.

Of the groups with a known relationship to the crowdfunding team, the one comprising patients (and their relatives) made the largest contribution to the funds (30.17%), followed by the groups of friends and family members, anonymous donors, and work colleagues (Table 2). Donors whose relationship to the crowdfunding team was unknown contributed the largest proportion of all funds raised (32.04%).

The median donation value per donor was significantly higher for the group of patients (and their relatives) (CHF 200, IQR 90) than for anonymous donors (CHF 50, IQR 80; *P*=.001) and for donors whose relationship to the crowdfunding team was unknown (CHF 87, IQR 140; *P*<.001).

Similar percentages of the funding came from donors who selected experiences (25.33%) and those who selected material rewards (25.93%), but most funding (48.74%) came from donors who forwent a reward (Table 2). In terms of the 12 individual rewards offered, most funding came from donors who selected the neurosurgical training in the skills lab (total CHF 7215), followed by those who selected the t-shirt (total CHF 4640), the work of art (CHF 4000), and the invitation to the scientific conference to be held at the end of the crowdsourcing competition (total CHF 3750). As far as it is possible to tell, donations came from 11 countries spread across three continents (Europe, North America, and Asia) (Table 2). The vast majority of funds came from donors based in Switzerland (82.33%), while the geographic location of 29.1% of donors (68/234) contributing 12.33% of the total donation sum was unknown.

**Table 2:** Characteristics of donations.

Characteristic			Donation (CHF^a^)		Value, %
**Relationship**					
	Patients and their relatives	17,820.00		30.17%	
	Unknown	18,922.69		32.04%	
	Friends	9288.00		15.73%	
	Colleagues	6028.00		10.21%	
	Anonymous	7001.00		11.85%	
	Total	59,059.69		100.00%	
**Type of reward**					
	Material	15,315.69		25.93%	
	Experiences	14,958.00		25.33%	
	Gift declined	28,786.00		48.74%	
	Total	59059.69		100.00%	
**Country of origin**					
	Switzerland	48,624.00		82.33%	
	Great Britain	1047.00		1.77%	
	Singapore	876.00		1.48%	
	Germany	272.69		0.46%	
	United States	259.00		0.44%	
	Greece	250.00		0.42%	
	Luxembourg	200.00		0.34%	
	Lebanon	160.00		0.27%	
	Austria	50.00		0.08%	
	Poland	20.00		0.03%	
	Liechtenstein	20.00		0.03%	
	Unknown	7281.00		12.33%	
	Total	59,059.69		100.00%	

^a^CHF: Swiss francs.

## Discussion

### Principal Results

Can neuroscientists use internet-based crowdfunding to kick-start new projects? The HORAO (from ancient Greek “to see with one`s mind”) campaign was able to reach its funding goal of CHF 50,000 within a month. Ultimately, we received 138% of the target (CHF 69,109) from 235 donors. This success is somewhat contradictory to the experiences reported in the scientific literature suggesting that crowdfunding can hardly yield sums larger than US $10,000 [4,10,11] and that similar to traditional funding agencies, donors are hesitant about supporting innovative projects [19]. What are the specific aspects of our campaign that could have contributed to its success?

### Contributors

The principle of crowdfunding consists of a large number of individuals contributing generally small amounts each. In our campaign, the group of donors whose relationship to the crowdfunding team was unknown contributed more funding than any of the other groups. However, the efforts made to reach out to the crowd were substantial. For instance, the financial cost of the sponsored 1-week online newspaper campaign would likely have exceeded the additional donations it brought in, if we had to pay for it. Hence, while we believe that reaching out to the crowd through news media and online portals will help to attain the funding goals, it is unlikely to be profitable if market prices have to be paid.

Patients and their relatives contributed the largest amount of funds. Given that patients and their relatives might one day themselves benefit from the success of our campaign, this finding comes as no surprise. Nevertheless, our results contradict a conclusion from a crowdfunding study on more general product marketing that compared the effectiveness of different types of appeal in raising money for product development [20]. This study found that product benefits to donors do not seem to be the most effective strategy. The incentive to donate money seems to obey different laws when it comes to life-threatening diseases such as cancer. Hence, we believe that identifying potential donors with a specific personal interest in the field of research is a key step toward ensuring the success of crowdfunding campaigns for medical research. Contacting specific interest groups, such as patient organizations with an interest in brain tumor treatment, might have attracted further donations. However, in our campaign, we refrained from contacting patients and their relatives directly and from encouraging them to donate in any way to avoid raising ethical concerns. Patients might otherwise get the false impression that failure to donate could entail negative consequences for their treatment.

Most of the funds raised during the first 2 weeks came from our existing network, consisting of not only friends, family, and work colleagues, but also patients and their relatives. However, unknown donors also started to contribute from the very first week. Our findings thus agree with the general observation that the initial donations in crowdfunding campaigns typically stem from the project creators’ own social networks [21,22]. However, our findings differ somewhat from those of an eHealth study on congenital heart disease, where unknown donors only started to contribute after observing some initial funding success [21]. We believe that the continuous preliminary campaign with attention-grabbing videos on social media in the weeks preceding the start of the crowdfunding campaign was responsible for the early buy-in by unknown donors. Hence, while it takes more effort to convince unknown donors to contribute, preliminary activities on social media might prime these potential donors to recognize the quality of the campaign, prompting them to donate earlier. The fact that at least 82% of our total funding came from donors located in Switzerland reconfirms the “home bias,” which is characteristic of crowdfunding transactions, that is, the crowd tends to support funders located in proximity to themselves [13]. Overall, our results confirm the importance of a large social network, which in this case included not only family and friends, but also patients, for success in obtaining funding.

### Timeline of Contributions and the Influence of the Media

Donations did not follow a linear pattern throughout the campaign. Instead, we observed a pronounced peak at the beginning, which is best explained by the actions of donors from our own network who had been primed for the campaign and donated once it started. In addition, we observed an increase in donation activity once the funding goal came within reach, presumably because this led hesitant donors to feel confident about the success of the campaign. The timeline of our donations replicates the U-shaped pattern according to which donors support campaigns preferably at their start and when funding approaches the target sum [23]. Hence, the dry period after the initial peak in funding requires additional effort. Our strategy of bridging the dry period with a second media campaign was successful to a certain extent.

It is difficult to assess the effect of the 14 media reports published on August 03 and August 04, 2017, since these coincided with the launch of the HORAO campaign. The article that appeared in both print and online versions during week three of the campaign resulted in a peak in contributions from both unknown and anonymous donors lasting several days, and thus, undoubtedly revived contributions from donors outside our own social network. In the literature, social media activity is reported to correlate with fundraising success, but the strength of this effect seems to depend on the type of campaign (creative, social, or entrepreneurial) [24]. To summarize, preparation of material to maintain a media presence is time-consuming and requires extra effort (eg, to create explanatory videos), but can help to bridge the slack period.

Since more than half of our funding goal (CHF 26,000) was contributed by the five largest donations and these were made by individuals from five different groups (all but the “unknown”), it is not possible to conclude that any one group would have been more critical to the success of our campaign than another. Most likely, our results demonstrate that the personal experience of affected patients and their relatives, in combination with their personal relationship to a physician launching a crowdfunding campaign, can be a powerful incentive to donate.

A study investigating the strategies and communication tools of reward-based crowdfunding campaigns identified the following three different paths to funding success, dependent on the type of project: “communicator,” “networker,” and “self-runner” strategies [12]. Self-runner projects catch the attention of the crowd, including the media, all by themselves, so rewards are hardly needed to attract backers. By contrast, communicator projects are rather weak by themselves, and their success depends on concerted efforts in online marketing and public relations as well as well-chosen rewards. Networker projects rely more on the personal network of the funders than communicator projects do, and rewards are essential to attract the personal network, while the information will then automatically flow to the general public, making web presence superfluous. Although a sufficient number of donors is a prerequisite for funding success independent of the path, social media activity is critical only in two of the three paths (communicator and networker). In these cases, the attention the crowd can pay to the projects is not sufficient at the outset, so regularly updated information is needed to maintain their interest. Moreover, rewards and web presence are thought to substitute for one another. It is conceivable that for patients and their relatives, our campaign was a “self-runner” project, whereas networking and regular communication were critical in attracting donors who did not have a personal link to the problem at stake.

### Selecting the Right Rewards

It is difficult to assess whether we selected the right rewards. None of the rewards clearly stood out in terms of funding success, and both experiential and material rewards contributed similarly to overall funding success. When choosing the number of rewards, we followed the recommendation in the literature, which had shown an inverted U-shaped relationship between the number of rewards offered in a crowdfunding campaign and the number of donors attracted (a moderate number of rewards is preferable to a lengthy list) [25]. Every third donor (35%) chose to forgo the reward, and half of our total funding was contributed by donors who did not claim any rewards. Our results seem to emphasize the importance of donors who do not require any incentives at all, presumably because our project represented an issue that is close to their heart.

### Crowdfunding Versus Traditional Funding

We assumed that because our project is largely application-oriented, it is not the type of project favored by traditional funding instruments. Moreover, as a strategically innovative and thus high-risk project, the chances of obtaining traditional funding are low [4]. For these reasons, we decided to address the crowd with our request. The ever-increasing competition for traditional (typically governmental) sources of project funding is the primary reason why project creators turn toward crowdfunding as an alternative way to obtain financing [4,11]. Crowdfunding offers the potential to reach a much broader and more diverse audience than traditional funding applications [10]. Besides the obvious benefit of obtaining funds, involving the crowd has the potential to bring more transparency into the mechanisms of science funding, as well as ideally building a lasting community and raising awareness [10].

### New Skills Needed

One of the main challenges of this project was to explain a specific scientific problem to the crowd, which requires a different communication strategy and different presentation skills from those needed to write grant applications for peer-reviewed funding [4]. Potential donors are likely to spend less time studying a project description and to have less pre-existing knowledge about this research area than peer reviewers, resulting in knowledge asymmetry [26]. Hence, the project description must be easily comprehensible and presented concisely in layman’s terms. The ability of scientists to engage with the crowd and build an audience around their project is one of the most quoted factors in successful scientific crowdfunding [4,26]. In addition to more factual written descriptions, short videos provide the narrative that captures the attention of a potential donor and helps to evoke emotions that trigger donations [10]. Coaching by crowdfunding professionals from the commercial crowdfunding platform (wemakeit) and by other scientists who had run crowdfunding campaigns was crucial in enabling us to identify and acquire the necessary skill set.

### Outlook

Research studies have attempted to establish crowdfunding as one form of entrepreneurial, technology, or science-related financing; however, little is known about the sustainability of crowdfunding projects. Crowdfunding can be split into the following two stages: funding and implementation [27]. A campaign’s success in reaching its funding goal does not necessarily guarantee success in project implementation. While in traditional funding processes, peer review is supposed to channel funding to the most promising projects, concerns have been raised about the scientific quality and sustainability of successfully crowdfunded science projects [5]. On the other hand, projects supported by the crowd might contribute to scientific outcomes more tailored to the needs of the general public than projects selected by the inherently biased opinion of a small number of peer reviewers. As opposed to the extensively studied funding stage, very little research has been published on the implementation of crowdfunding projects [27]. Further studies are needed to investigate the performance of scientific and technological ventures after successful crowdfunding, as well as the impact of crowdfunding on science in general.

### Limitations

The external validity of our results may be limited. Campaign success depends on the size of the team’s social network and on the specific goal. Cancer-related issues may trigger stronger emotions than other neuroscientific topics, which might have contributed to the success of the present campaign. Some benefits, such as promotion on the news portal and support from the foundation, are because of the project’s novelty. In addition, the success of our campaign should not be seen as proof of the scientific value of our endeavor.

## Appendix

Multimedia Appendix 1 Promotional video from the crowdfunding website.

## Abbreviations

CHF: Swiss francs
